# The Need for an Alternative to Culling Day-Old Male Layer Chicks: A Survey on Awareness, Alternatives, and the Willingness to Pay for Alternatives in a Selected Population of Dutch Citizens

**DOI:** 10.3389/fvets.2021.662197

**Published:** 2021-06-17

**Authors:** Elske N. de Haas, Eva Oliemans, Maite A. A. M. van Gerwen

**Affiliations:** ^1^Animals in Science and Society, Faculty of Veterinary Medicine, University of Utrecht, Utrecht, Netherlands; ^2^Department of Animal Welfare, Flanders Research Institute for Agriculture, Melle, Belgium; ^3^Centre for Sustainable Animal Stewardship (CenSAS), Faculty of Veterinary Medicine, University of Utrecht, Utrecht, Netherlands

**Keywords:** chicken, in-ovo sexing, willingness-to-pay, dual-purpose chicken, male layer chicks, day-old chicks

## Abstract

A concerning by-product of producing laying hen chicks are the hatched male layer chicks. As a consequence of their inability to lay eggs, these male chicks are culled as day-old chicks in the hatchery. To find an alternative for this ethical dilemma (generally), three alternatives are under study, namely, *in ovo* sex determination, using dual-purpose breeds, and the rearing of layer cockerels. In order to assess the awareness of this practice and preference for one of the alternatives, we conducted an online survey of the Dutch public. Most of the 259 respondents completing the survey were highly educated woman (HEW, *n* = 143) versus others (REST, *n* = 86). The questionnaire was divided into six topics: (1) general knowledge of the poultry industry, (2) awareness of culling male layer chicks (CMC), and (3) its acceptability, (4) alternatives to CMC, (5) willingness to pay (WTP) for eggs without CMC, and (6) WTP for cockerel meat. Awareness about CMC was 52%, and its acceptability was rejected by 78% (HEW) and 67% (REST). The level of acceptability increased when more salient facts were given, and almost all respondents agreed that an alternative was needed (90% HEW, 84% REST). For both groups of respondents, more than 50% preferred *in ovo* sex determination over keeping the current practice or using dual-purpose breeds or male layers. Furthermore, the majority of respondents were willing to pay more than double the price for eggs without CMC being involved. Roughly 40% would not buy processed cockerel meat burgers, most likely due to their vegan or vegetarian diet. Of the remaining respondents, half were willing to pay the current price or 1 euro more for processed cockerel meat burgers. The most important factors when buying poultry meat or eggs without CMC were food safety, animal friendliness (welfare), and the environment; price was the least important factor. Despite the skewed respondents' background, the results of our survey show that consumers are willing to pay more for poultry products that do not require culling day-old male chicks.

## Introduction

A moral dilemma within the egg production industry is the culling of day-old male layer chicks (1). In order to produce eggs, laying hens are bred. The “brothers” of these bred laying hens are immediately culled at hatch, as male layers do not lay eggs and are deemed unqualified for the production of chicken meat. Culling of day-old male layer chicks (CMC) is noted to be of animal welfare concern within the egg production chain in the EU, and alternatives are seeked for this practice (2). Yearly, at least 7 billion male chicks are culled at hatch (3). The method for CMC in the United States of America is by shredding or maceration, leading to immediate death (4). The most common used method for CMC in Dutch hatcheries, however, is by asphyxiation with a high percentage of carbon dioxide (CO^2^) (5). After asphyxiation, deceased chicks are often directly frozen and used as food for snakes (6), reptiles, birds of prey, or other types of animal feed. The CMC raises ethical concerns with regard to animal welfare (7) and the number of animals culled (8) and has led to political debate on CMC (1, 9). Especially within Germany, this issue has been raised (10). The ethical dilemma in the egg production industry likely has led to bans on CMC in France and Germany from 2021.

As a result of the political and societal disapproval toward CMC, research is aimed at finding alternatives for this practice (3). There are three alternatives that are currently used to varying degrees in the egg industry, while others are still being further refined. The three alternatives are *in-ovo* sex determination (3), keeping the roosters of dual-purpose breeds for meat production (11–14), and rearing roosters of layer breeds for processed meat products (15).

*In-ovo* sex identification is the determination of the sex of the embryo while in the egg (16, 17). With *in-ovo* sex identification, male embryos can be detected and then excluded from further development prior to or during incubation. Different sex determination techniques exist that differ in the level of invasiveness, which can influence hatchability (18, 19) [see (3), for review]. In the Netherlands, a Biotech company developed an *in-ovo* sexing technique using biomarkers. On day 8 of incubation, the egg is penetrated by a needle and a small amount of biological material is removed to determine the sex of the embryo with the use of a specific biomarker (https://inovo.nl/solutions/in-ovo-egg-sexing). A similar method is described by Weismann et al. (16, 17), where *in-ovo* sexing takes place by analyzing estrone sulfate in the allantoic fluid in the incubated eggs (20). Male embryos have lower levels of estrone sulfate compared to females, and sexing accuracy above 98% could be attained with this technique (16). The technique requires penetrating the egg, which could be a risk for survival due to infection risks. This *in-ovo* technique was shown to reduce hatching rate and rearing performance of layer females, which were sampled at day 9 of incubation as opposed to an unsampled control group, but no effect on laying performance was noted (17).

Another technique of *in-ovo* sex determination is by optical imaging methods (3) such as reflectance (21), infrared (22), and Raman spectroscopy (23–26). By penetrating the egg with a CO^2^ laser and using spectroscopy on the embryo, the sex of the embryo can be determined based on different absorption spectra (3). This method is less invasive than removing biological material, but it still requires opening the eggshell. Reflectance spectroscopy allows sex identification with 90% accuracy when performed at day 10 of incubation (21). Fourier transform infrared spectroscopy is possible in non-incubated eggs because it is based on assessing the blastoderm cells in the germinal disc (22). For these optical imaging methods to assess sex, the eggshell needs to be opened, which is a risky avenue as the dimension of the hole in the eggshell reduces hatching rate (23). Through the hole in the eggshell, near-infrared fluorescence can identify the sex of the embryo [for details, see (24–26)]. These imaging techniques are preferred over assessing biomarkers from the allantoic fluid due to their low risk of contamination, being contact free and fast, and can be made automatic. At what age of embryonic development the *in ovo* techniques are applied could be an important aspect that may help people determine whether or not this technique is chosen as an alternative to CMC. A non-penetrating *in-ovo* sex identification method is *via* genetic marking of sex chromosomes (27, 28). Cockerels possess two Z sex chromosomes (i.e., they are homogametic), while females have one Z and one W sex chromosome (i.e., they are heterogametic). Genetically engineering the females is studied by Doran et al. (29) and Quansah et al. (30). They marked the breeding hens' Z chromosome with a fluorescent protein. In the germinal disc, sex-specific patterns could be determined to assess sex in non-incubated eggs (29).

Another alternative to CMC at hatch is by rearing the male layer chicks for their meat, which in some studies has been chosen as an acceptable alternative (15, 31). Layer chickens are bred for the production of eggs; therefore, both sexes are lean, have a high feed intake, and are of less mature body weight compared with broilers specifically bred for high growth rate and muscle mass (32). Furthermore, the carcass qualities of layer males are quite different compared to broiler chickens (33). Layer males would be kept up to 18 weeks of age in order to reach a body weight of around 2 kg (9). Broiler chickens (male or female) can grow to 2 kg in 33 days and grow out to 5 kg in 70 days depending on the genotype used (2). Body parts such as the breast muscle or thighs of broilers are used as meat products, but the meat of layer cockerels needs to be processed into different products, such as burgers (15). The meat products obtained from the layer cockerel are of lesser quality and quantity (34), thus more costly (i.e., less economical), and less sustainable compared with broiler meat because of the higher cost of feeding and housing these birds for a longer period of time and further processing demands. At present, only a limited number of farmers in the Netherlands use this avenue likely due to the economic and sustainable issues.

By using dual-purpose chickens, the cockerels are slightly more suited for meat production as the conventional layer genotypes (35). The meat from dual-purpose breeds is more comparable to broiler meat in taste and texture (14). Dual-purpose roosters can become heavier than layer roosters (i.e., 3 kg over 2 kg) (9). However, compared to the conventional layer or broiler genotypes, dual-purpose chickens produce fewer eggs and have lesser meat production being less economical and sustainable, as they need 14 weeks to attain 2 kg over 5.5 weeks for the common broiler (9). This means that dual-purpose chickens need 9.5 weeks longer to get the same body weight as the common broiler. The dilemma of CMC—i.e., there is no ideal alternative based on animal welfare, economics, ethics, and sustainability—makes it difficult to choose an alternative; therefore, we focused on animal welfare in this study.

Research has also suggested that alternative chicken meat products would not be fully accepted by Dutch society or the poultry industry (9, 36). To assess the public views, awareness, and acceptance of cockerel meat products, several surveys and workshops (3) have taken place in the EU [Germany: (10, 37–39); Switzerland: (40)]. Based on surveys in the Netherlands, public awareness on this topic increased from 42% in 2007 to 55% in 2015 (9, 36). The preference for *in ovo* sexing and keeping layer males instead of CMC was compared by Gremmen et al. (36) in an online survey conducted in the Netherlands in 2015. Neither one of the approaches was fully accepted. Leenstra et al. (9) looked at the public view to nine alternatives to CMC and accepting CMC. As the practice of CMC and the pros and cons of the alternatives are likely unknown to the participants Leenstra et al. (41), Leenstra et al. (9) provides background information *via* video footage. In this study, *in-ovo* sexing of the fresh egg and keeping a dual-purpose chicken were chosen as most preferable to CMC. More than half the respondents who choose an alternative to CMC were willing to pay an additional 5–10 euro cents per egg from dual-purpose breeds or eggs obtained using the *in ovo* method (9). These results give the suggestion that Dutch consumers of poultry products became more aware of the practice of CMC over the years (36) and are inclined to pay more for poultry products where CMC is excluded. These surveys in the Netherlands were conducted more than 5 years ago, and therefore, we wanted to assess the current public awareness of CMC and their preferred alternative (*in-ovo* sexing, rearing male layers or dual-purpose chicken) and their willingness to pay (WTP) for that alternative. Slightly similar to the study by Leenstra et al. (9), we wanted to provide information on the poultry industry to the participants in order to assess their change in acceptance of CMC. Our goal was to reach an evenly distributed population of respondents with different demographic background, however our respondents mostly fitted a highly educated subset and thus our results are limited to those people of the Dutch population.

## Materials and Methods

An online questionnaire was created in Dutch using Qualtrics (see Additional file 1). Respondents could fill in the questionnaire from July 3 until August 1, 2020. Only questions were provided, and no video or other footage was used. Questions were formulated to obtain information on six different topics by order of appearance in the survey: general knowledge on the poultry industry, of CMC, WTP for eggs without CMC, WTP for cockerel meat without CMC. Each topic was introduced so as to provide the respondents with our aim for the questions and—if needed—to establish background needed for answering the questions. In some cases, this text provided respondents with answers to the previously asked questions (Table 1). The survey was made up of six parts. Part 1: Eight questions regarding the poultry industry in the Netherlands. First two questions with multiple-choice answers, followed by six questions with a *true/false/I do not know* answer option. Part 2: Four questions regarding the acceptance of CMC, following were six questions on acceptance of keeping chickens for food and the alternatives for CMC, which could be answered based on a Likert agreement scale (1 = strongly disagree, 5 = strongly agree). Part 3: Ranking the alternatives according to preference (1 = most preferable, 4 = least preferable). Part 4: Seven questions on factors playing a role in the choice for poultry products on a Likert importance scale (1 = not important at all, 5 = very important). These factors included price, environment, availability of the product, food safety, naturalness (not specifically defined), animal friendliness/animal welfare, taste of the product, and feasibility of the alternative. Part 5: Six questions with multiple-choice answers regarding choice for poultry products and WTP for eggs and cockerel meat without CMC. First, respondents were asked which type of eggs or poultry meat they typically buy. Next, they were asked how much they are willing to pay for eggs and cockerel burgers without the CMC and whether a label on the product showing that the product is produced without CMC assists in their choice for these egg or poultry meat products. To determine if there had been a change in acceptability of CMC prior to and after completing the questionnaire, respondents were again asked on their agreement for CMC using the Likert agreement scale. Part 6: Eight questions regarding sociodemographics and personal information such as sex, age, highest level of education, annual income, province of residence; diet-choice; owning of pets; and whether they donate money to charity. The invitation to participate in this questionnaire was distributed through social media (Facebook, Twitter, and LinkedIn) *via* the personal accounts of all three authors. This has resulted in a biased background in the respondents. Only fully completed questionnaires were used for analysis (i.e., 259 out of 372).

**Table 1:** Additional information given in the survey on culling day old male layer chicks.

**Content of information provided**	**When was information provided**	**Prior to which questions—paragraph content**
The poultry industry is specialized. Chickens for meat and chickens for eggs. This survey is aimed to test your knowledge and opinion on eating cockerel meat	At the start of the questionnaire	1–8.Paragraph 1. Knowledge on the poultry industry
In the Netherlands, 90,000,000 chickens are kept yearly. 45,000,000 chickens for meat and 45,000,000 chickens for eggs. Because cockerels of layer chicken do not lay eggs they are culled at the hatchery. Once chicks hatch sex is determined manually by a specialist. Once the sex is determined to be male, male layer chicks are culled via CO_2_ asphyxiation. Yearly 45,000,000 male layer chicks are culled.The culled chicks are used for zoos, reptiles and birds of prey and other animals.	After first paragraph of questions regarding the numbers of chicken in the Netherlands, which day old male chicks are culled (broiler and layer or only layer), the sexing method, the method of culling and what happens with the culled male layer chicks.	9–12Paragraph 2. Acceptance of culling day old chicks
Culling day old chicks is causing a discussion on animal and ethics. Research is being conducted on alternatives for culling day old layer males. In this survey we look at three alternatives. We consider herein the perspective of keeping the animals under the highest welfare conditions.1. Keeping layer males for special cockerel meat products.2. Double purpose chickens for keeping the cockerels for meat and the hens for egg production.3. Sex determination of the embryo in the egg. With a needle fluid is being taken from the embryo which is used to determine the sex. Male embryos will be excluded from further development and used in animal feed.	After paragraph 2. Acceptance of culling day old chicks.	13Paragraph 3. Acceptance of alternatives to culling day old male layer chicks19Paragraph 4. Preference of alternatives to culling day old male layer chicks
Broiler chickens are slaughtered at 6 weeks of age. When cockerels of layer breeds will be kept for meat this takes ~15 weeks, because they take longer to grow. The feed and care costs are higher as compared to broiler chicken. This makes the meat of these layer cockerels more expensive than meat of broiler chicken. Meat from cockerel layers is less tender than meat from broiler chicken, and therefore the meat is processed into sausages or burgers.	After paragraph 2. Acceptance of culling day old chicks.	13–18Paragraph 3. Acceptance of alternatives to culling day old male layer chicks19Paragraph 4. Preference of alternatives to culling day old male layer chicks
A double purpose chicken is a chicken breed which can be used for meat and eggs. The meat of double purpose chicken is more expensive because they do not grow as fast at the broiler chicken. The feed and care costs for double purpose chicken are higher as compared to the broiler chicken.	After paragraph 2. Acceptance of culling day old chicks.	13–18Paragraph 3. Acceptance of alternatives to culling day old male layer chicks19Paragraph 4. Preference of alternatives to culling day old male layer chicks
Layer cockerel meat is mainly being used for sausages and burgers. This is currently the cockerel burger from layer cockerels available in one specific supermarket chain. 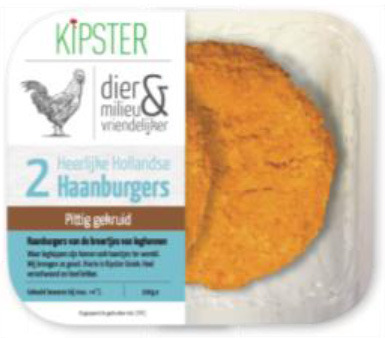	After question 31 in Paragraph 6. Willingness to pay for chicken products	32

### Statistical Analysis

The data were analyzed using Statistical Package for the Social Sciences (SPSS, version 23). Descriptive analysis was performed on the sociodemographic information of the respondents. We noticed a high number of women with a high education level originating from two specific provinces out of 12 provinces in the Netherlands as can be seen in Table 2A. The data were divided into two subsets (Tables 2A,B), so that any statistical analysis would not be wrongly attributed to the large group of highly educated women (HEW). That is, more than half of the respondents were HEW, subset 1 (*n* = 143). Subset 2 contained the rest of the respondents (REST: *n* = 86). Our aim was not to compare subsets but to limit incorrect conclusions on demographics as a consequence of the background of HEW skewing the dataset. In order to have an equal number of respondents in specific categories, we combined provinces together as follows: South NL: Limburg and Noord-Brabant; Urban South-West: Noord-Holland and Zuid-Holland; North NL: Overijssel, Drenthe, and Groningen. The same was done for age group 51–60 and 60+ in subset 1. We excluded one respondent below 17 years old and two in the category 17–21 due to low sample sizes.

**Table 2A:** Socio-demographic characteristics of subset 1: Highly educated women.

	**Sample (*n* = 143)**	**Sample (%)**	Dutch population^*^ %
**Age**			
21–30	39	27.3	12.7
31–40	38	26.6	12.2
41–50	31	21.7	13.1
>50	35	24.5	14.5
**Income (per year, per household)**			
Low (< € 29.999)	37	25.9	60.7
Middle (€30.000–€49.999)	37	25.9	31.9
High (>€50.000)	41	28.7	7.4
Unknown	28	19.6	
**Province**			
South NL	20	14	21.2
Urban South-West	39	27.3	37.8
Utrecht	36	25.2	7.8
North NL	20	14	14.8
Gelderland	28	19.6	12.0

*All 143 respondents in subset 1 were women with a high education level (University degree)*

* *Dutch Central Bureau for Statistics (CBS statline), accessed August 2020. Income: Low: < € 29.999; Middle: €30.000–€49.999; High: >€50.000 (per year per household). NL, The Netherlands*.

**Table 2B:** Socio-demographic characteristics of subset 2: REST.

	**Sample (*n* = 86)**	**Sample (%)**	Dutch population^*^ %
**Sex**			
Men	65	75.6	49.6
Women	21	24.4	50.3
**Education**			
Middle	48	55.8	36.7
High (University)	38	44.2	32.3
**Age**			
21–30	17	19.8	12.7
31–40	21	24.4	12.2
41–50	12	14.0	13.1
51–60	18	20.9	14.5
60+	18	20.9	25.3
**Income (per year, per household)**			
Low (< € 29.999)	22	25.6	60.7
Middle (€30.000–€49.999)	21	24.4	31.9
High (>€50.000)	31	36.0	7.4
Unknown	12	14.0	
**Province**			
Urban South-West	28	32.6	37.8
Utrecht	20	23.3	7.8
South NL	12	14.0	21.2
Gelderland	26	30.2	12.0

* *Dutch Central Bureau for Statistics (CBS statline), accessed August 2020. NL, The Netherlands*.

First, descriptive analysis on knowledge levels of the Dutch poultry industry (true/false answer options for part 1) was performed. Likert scale data were analyzed as frequencies of respondents adding agree + strongly agree and adding disagree + strongly disagree together and comparing these frequencies with a chi-square test. Change in acceptability of CMC was calculated for the subsets by comparing the response to the first and second questions on acceptability (in the beginning and at the end of the survey). To test which alternative was preferred, the overall percentage of first preference was calculated and analyzed with a chi-square test. The relative importance of the factors influencing choice of product was assessed with a Wilcoxon signed rank test. The WTP for eggs and for meat without CMC was assessed with a Wilcoxon rank sum test. A chi-square test was used to determine whether income levels were affecting the price respondent were willing to pay for eggs and for meat with or without CMC.

## Results

The sociodemographic data of both subsets can be seen in Table 2A (HEW) and Table 2B (REST). Under the respondents in subset REST, there were more men than women (76 vs. 24%), more people with a high income compared to middle or low (36 vs. 24% and 26%). In both subsets, the province of Utrecht was overrepresented compared with the Dutch percentage (Tables 2A,B).

### Awareness Regarding the Practice of Culling Day-Old Male Layer Chicks and General Knowledge of the Poultry Industry

**Figure 1:**
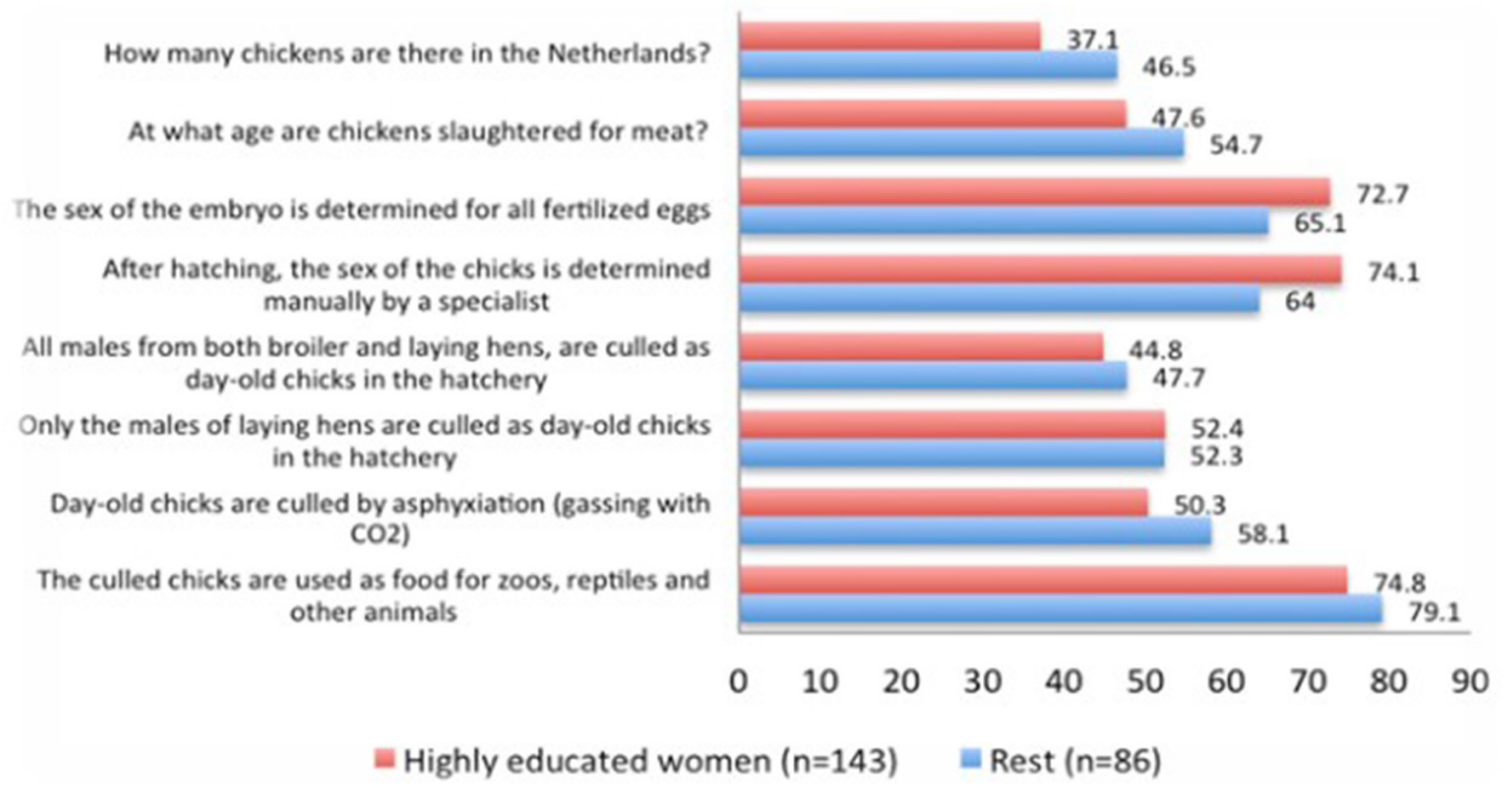
Percentage of respondents correctly answering general knowledge questions/statements on the poultry industry, its practices and the culling of day-old males correctly.

For both subsets, roughly 52% knew about the practice of CMC (Figure 1). The majority of respondents were aware that culled chicks are used as animal food (HEW: 74.8%; REST: 79.1%). Roughly half of the respondents knew about the method of culling (HEW: 50.3%; REST: 58.1%) and that only male layer chicks are culled and not male broiler chicks (HEW: 44.8%; REST: 47.7%). The majority of respondents knew that sex determination of chicks takes place after hatch (HEW: 73%; REST: 65%) by a specialist (HEW: 74%; REST: 64%). Regarding the general knowledge on the Dutch poultry industry, <50% of the respondents answered all questions correctly (HEW: 34.4%; REST: 32.6%). The most incorrect answers were given on the question regarding the chicken population in the Netherlands (HEW: 37.1% and 46.5%).

### Acceptance Regarding the Poultry Industry, the Practice of Culling Day-Old Male Layer Chicks, and Alternative for Culling Day-Old Male Layer Chicks

See Table 3 for mean and median levels of the respondents' acceptance levels on 10 statements regarding CMC. All five questions regarding accepting the practice of CMC were disagreed (1) or strongly disagreed (2) (min–max mean levels: HEW: 1.72–2.02; REST 2.13–2.45). This was also significantly shown by the percentage of respondents disagreeing with CMC in HEW (78.8%) and REST subset (67.3%), *X*^2^ = 3.65, *P* = 0.05. A smaller percentage of respondents disagreed with CMC as unavoidable (HEW: 44.8%; REST: 26.7%; *X*^2^ = 7.03, *P* < 0.001). Keeping chickens for food tended to be accepted based on high Likert scale levels (1 = strongly disagree, 5 = strongly agree: HEW: 3.52; REST: 3.92). Roughly half of the respondents agreed with keeping chicken for food production (51%: HEW; 45.3% REST). Very high levels of agreement were seen regarding finding an alternative for CMC (HEW: 4.49; REST: 4.22), with highest levels of acceptance for *in-ovo* sex determination (3.85 for both subsets).

**Table 3:** Mean and median outcomes for 10 agree/disagree questions rated on a Likert scale (1 = strongly disagree, 5 = strongly agree) regarding the acceptability of practices in the poultry industry.

**Questions regarding practices in the poultry industry**	**Highly educated women (*****n*** **=** **143)**		**REST (*****n*** **=** **86)**	
	**Mean**	**Median**	**Mean**	**Median**
The culling of day-old male layer chicks is a good solution.	1.72	1	2.24	2
There is no problem with culling day-old male layer chicks.	1.66	1	2.13	2
The culling of day-old male layer chicks is inevitable.	1.87	2	2.37	2
Day-old male layer chicks may be culled	2.07	2	2.45	2
Because day-old male layer chicks have another use, this eliminates the need for an alternative to CMC.	2.01	2	2.41	2
Chickens can be kept for food production.	3.52	4	3.92	4
An alternative is needed for the culling of day-old male layer chicks.	4.49	5	4.22	4
The use of dual-purpose breeds is a good alternative to the culling of day-old male layer chicks.	3.41	4	3.24	3
Rearing male layers is a good alternative to the culling of day-old male layer chicks.	3.51	4	3.47	4
In-ovo sex determination is a good alternative to the culling of day-old male layer chicks.	3.85	4	3.85	4

*Likert scale used here: 1 = strongly disagree, 2 = disagree, 3 = neutral, 4 = agree, 5 = strongly agree*.

### Change in Acceptance Levels of Culling Day-Old Male Layer Chicks Prior to and After the Questionnaire

**Figure 2:**
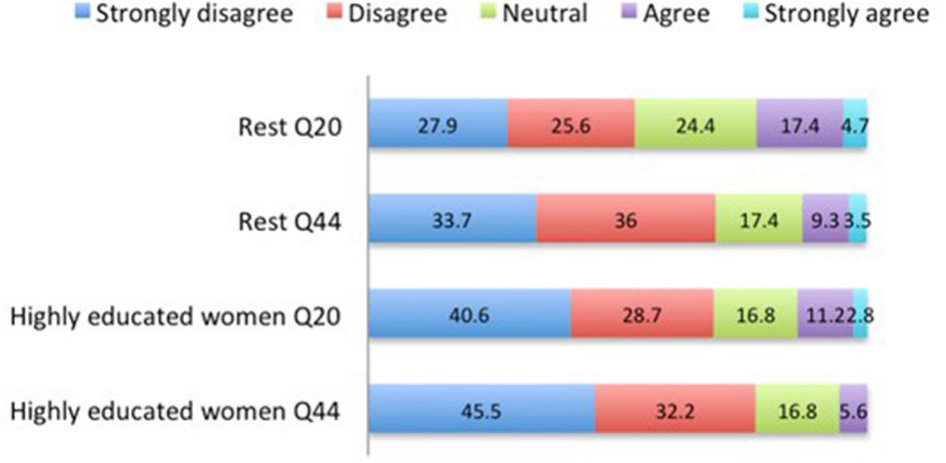
Percentage of respondents' agreement on culling day-old male chicks at the beginning of the survey and at the end. Q20 “Day-old males may be culled”; Q44 “In this survey you received information about the poultry industry and its practices. To what extent do you agree that day cockerels are killed?” Highly educated women (*n* = 143), Rest (*n* = 86).

For both subsets, the percentage of respondents who disagreed with the practice of CMC increased, although not significantly (*X*^2^ = 1.55, NS; Figure 2). At the start of the survey, for HEW, 40.6% strongly disagreed to accepting CMC vs. 45.5% after the survey. At the start of the survey, for REST, 27.9% strongly disagreed with accepting CMC vs. 33.7% after the survey.

### Preference for Alternatives for Culling Day-Old Male Layer Chicks

**Figure 3:**
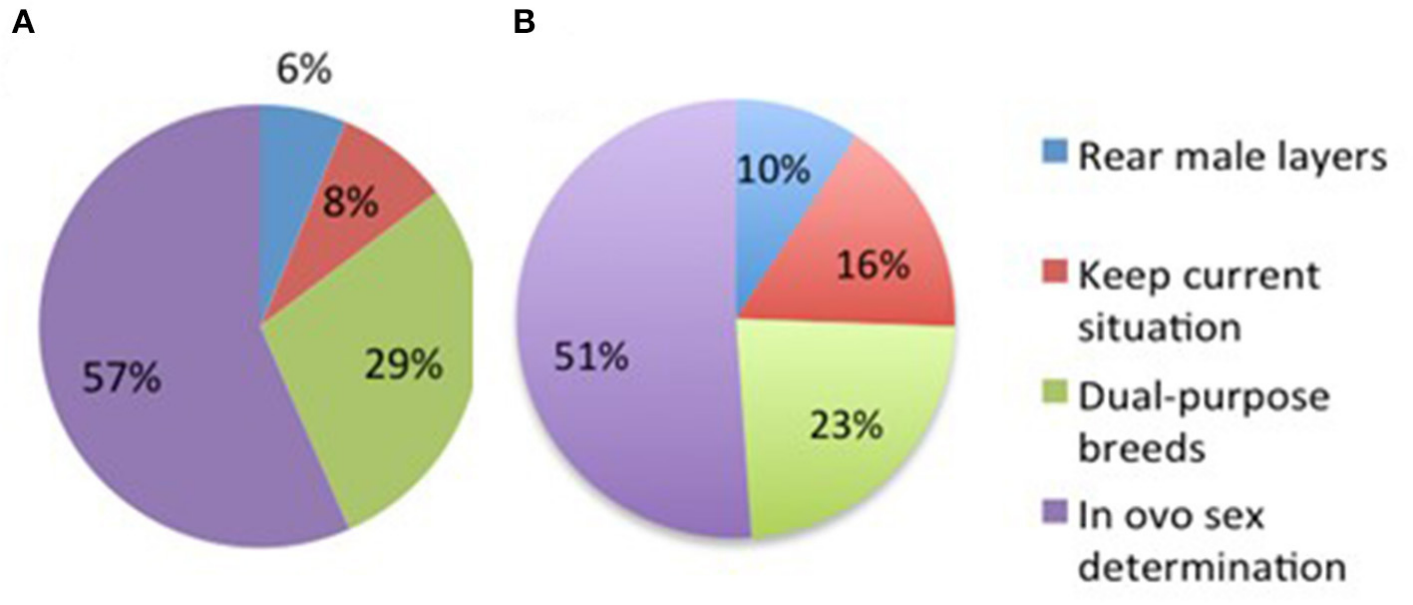
Percentage of respondents' preference of alternative to culling day-old male chicks **(A)** Highly educated woman (*n* = 143) and **(B)** Remaining group of respondent (*n* = 86).

Respondents were asked to arrange the alternatives to CMC in order of preference. The majority of the respondents chose *in ovo* sex determination as first preference as alternative to CMC (HEW: 57%; REST: 51%; Figure 3), followed by keeping dual-purpose breeds (HEW: 29%; REST: 23%). Rearing layer males were scored the least as first preference (HEW: 6%; REST: 10%), even lower than keeping the current situation (HEW: 8%; REST: 16%). For effects due to income, education level, awareness, and change in acceptability, see Table 4. More respondents with a higher level of awareness (*X*^2^ = 15.08, *P* < 0.05) and higher income (*X*^2^ = 19.15*, P* < 0.05) chose *in-ovo* sex determination as first preference.

**Table 4:** Chi-square test-statistics representing effects of income levels, education level, awareness levels and change in acceptance of culling day-old male layer chicks on alternatives for this practice.

	**Rear male layers**		**Accept current situation**		**Dual-purpose breeds**		**In-ovo sex determination**	
	**HEW**	**REST**	**HEW**	**REST**	**HEW**	**REST**	**HEW**	**REST**
Income level	*X*^2^ = 8.56 *P* = 0.48	*X*^2^ = 12.12*P* = 0.21	*X*^2^ = 13.35 *P* = 0.147	*X*^2^ = 5.02*P* = 0.83	*X*^2^ = 11.88 *P* = 0.22	*X*^2^ = 12.10*P* = 0.208	*X*^2^ = 3.47 *P* = 0.94	***X***^**2**^ **=** **19.15***P* **=** **0.02**
Education level	N.D.	*X*^2^ = 1.92*P* = 0.59	N.D.	*X*^2^ = 3.61*P* = 0.31	N.D.	*X*^2^ = 2.74 *P* = 0.434	N.D.	*X*^2^ = 0.72*P* = 0.87
Awareness	*X*^2^ = 7.02 *P* = 0.32	*X*^2^ = 5.55*P* = 0.48	*X*^2^ = 6.68 *P* = 0.34	*X*^2^ = 6.92*P* = 0.33	*X*^2^ = 7.77 *P* = 0.26	*X*^2^ = 10.36*P* = 0.110	*X*^2^ = 11.70 *P* = 0.07	***X***^**2**^ **=** **15.07***P* **=** **0.02**
Change in acceptability	*X*^2^ = 1.45 *P* = 0.69	*X*^2^ = 0.19*P* = 0.98	*X*^2^ = 4.07 *P* = 0.25	*X*^2^ = 0.94 *P* = 0.82	*X*^2^ = 4.19 *P* = 0.24	*X*^2^ = 3.66 *P* = 0.30	*X*^2^ = 2.21 *P* = 0.53	*X*^2^ = 5.72*P* = 0.13

*HEW, subset highly educated woman; REST, remaining subset; N.D., not determined only one education level in this subset. Bold represent significant factors*.

### Factors Influencing the Choice of Alternatives for Culling Day-Old Male Layer Chicks

The most important factor for choice of alternatives for CMC is food safety in both subsets, followed by animal friendliness, the environment, naturalness, taste, feasibility, availability of the product, and price (Table 5; HEW: *X*^2^ = 273.7, *P* = 0.000; REST: *X*^2^ = 152.03, *P* = 0.000). No relationship was found between whether price was the most important factor and income level (HEW: *X*^2^ = 15.89; REST: *X*^2^ = 27.9, NS).

**Table 5:** Ranking of determining factors when buying eggs or poultry meat (rated on Likert scale 1–5).

	**Highly educated women (*n* = 143)**	**Rest (*n* = 86)**
Food safety	5.86	5.84
Animal friendliness	5.62	5.48
Environment	5.18	4.87
Naturalness	4.76	4.76
Taste	4.43	4.70
Feasibility	4.17	4.48
Availability of the product	3.15	3.25
Price	2.83	2.62

*Factors were not explicitly defined. Highly educated women Friedman's test: X^2^ = 273.769, df = 7, P = 0.000; Rest Friedman's test: X^2^ = 152.024, df = 7, P = 0.000. Post hoc analysis with Wilcoxon signed-rank tests. The higher the value, the more important respondents find this factor*.

### Willingness to Pay for Eggs Without the Practice of Culling Day-Old Male Layer Chicks

WTP more for eggs without CMC was seen in 41.9% of respondents vs. 45.3% who is willing to pay equal to the general costs for 10 eggs. There is a significant difference in WTP for the general costs for 10 eggs and the extra costs for 10 eggs without CMC (REST: *Z* = −4.134, *P* = 0.000). In the HEW, respondents were willing to pay more for 10 eggs without CMC (*Z* = −7.368, *P* = 0.000). Here, 62.9% of HEW are willing to pay more for eggs without any culling, whereas 26.6% are willing to pay equal to the general costs for 10 eggs. Only 10.5% of HEW and 12.8% of the remaining respondents are not willing to pay more for eggs without any culling.

### Willingness to Pay for Chicken Meat Without the Practice of Culling Day-Old Male Layer Chicks

**Figure 4:**
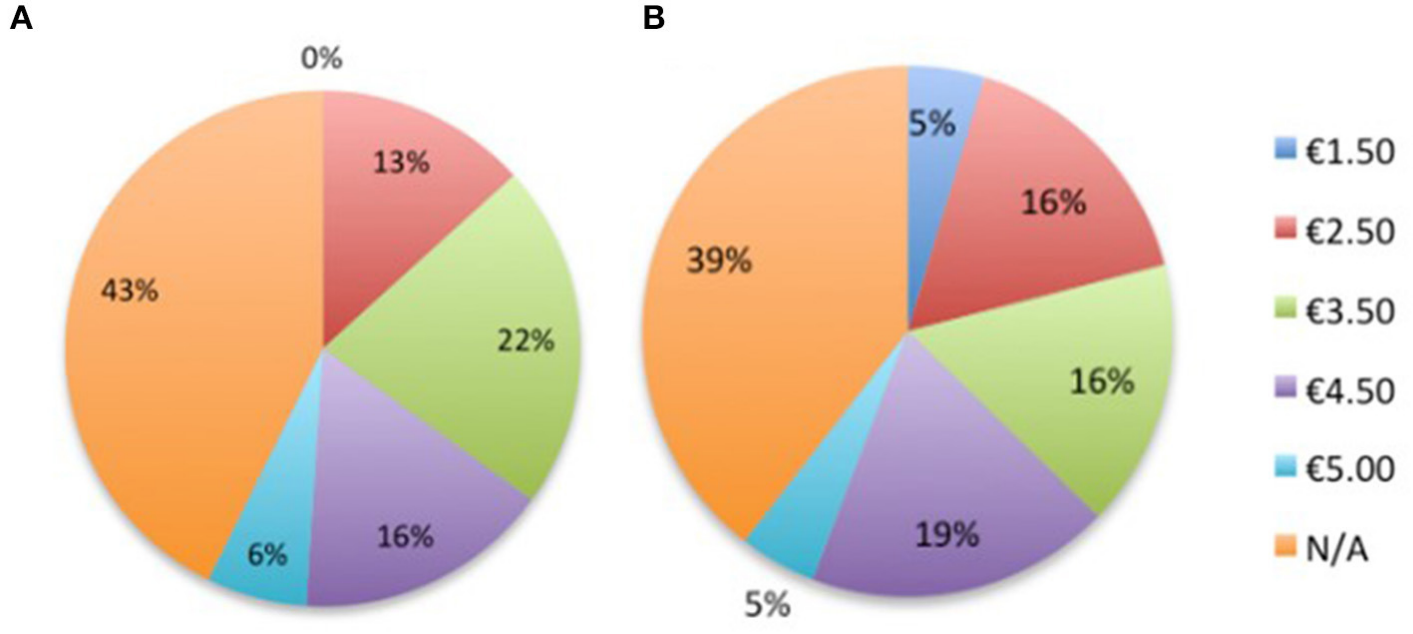
The percentage of respondents and their willingness to pay an absolute price for cockerel meat burgers in **(A)** Highly educated woman (*n* = 143) and **(B)** Remaining group of respondent (*n* = 86).

In both subsets, a large group of respondents answered not applicable to the question WTP cockerel burgers (HEW: 43%; REST: 39%), see Figure 4. Of this N/A group, 77% had a special diet in HEW, and 52.9% in REST. WTP (and effects of income levels) was, therefore, only assessed for the remaining respondents. The WTP for two cockerel meat burgers was €3.50 in HEW (21.7%; mean: 4.07; median: 4.00) and €4.50 in REST (18.6%; mean: 3.86; median: 4.00). The set price for cockerel burgers was €2.50, indicating WTP 1 or 2 € on top of the original price. Income levels did not affect respondents' WTP for 10 eggs with or without culling for both subsets (HEW: general cost: *X*_2_ = 22.064; NS, no culling: *X*_2_ = 17.390, NS, and REST: general cost: *X*_2_ = 19.78, NS; no culling: *X*_2_ = 24.097, NS). No relationship was seen between income level and the WTP for cockerel meat in both subsets (HEW: *X*_2_ = 16.533, NS; REST: *X*_2_ = 16.625, NS). WTP for cockerel meat was affected by whether the product contained a label “produced without CMC” (HEW: *X*^2^ = 48.17, *P* = 0.03; REST: *X*^2^ = 22.25, *P* < 0.05). No relationship was found between income level and WTP for cockerel meat (HEW: *X*^2^ = 16.53, NS; REST: *X*^2^ = 16.63, NS).

### Comparison to Other Surveys

When comparing our results with other surveys on CMC (Table 6), we note that the percentage of respondents being aware of CMC was similar to the Dutch study in 2015 (52 vs. 55%) but lower to a German study in 2016 (70%) and higher than the Dutch study in 2011 (42%) and a Swiss study (25%). Percentage of respondents not accepting CMC or indicated that there is a need for an alternative to CMC was relatively high (67.3–78.8%) compared to the previous Dutch studies in 2011 (58%) and 2015 (47%). In a recent German study with 482 respondents, a need for an alternative was chosen by 89%. No direct comparison could be made between studies regarding preferences for alternatives to CMC, as the presented alternatives slightly differed, i.e., more information on *in ovo* techniques was given (39) or the focus was on dual-purpose chicken (10, 40), and rearing male layers was not included (9, 10, 39, 40). However, *in-ovo* sex determination as an alternative to CMC was preferred in more than 50% of our respondents, which was higher than in any other study except for a study in Germany in 2018 (75%).

**Table 6:** Surveys on culling day-old male layer chicks.

**Reference by recent publication date**	**Date of survey and country where survey was conducted**	**Type of survey**	**Type of respondents, sample size, incentive to participate**	**Percentage of respondents' being aware of the practice of culling day old male layer chicks (CMC)**	**Percentage of respondents' not accepting/need for an alternative to CMC**	**Percentage of respondents' first preference for alternatives to CMC focusing on keeping dual purpose (DP) chicken; rearing layer males and in-ovo sex determination**
Current study	July-Aug 2020	Online survey 34 questions	259^*^, (in)directly within personal network of authors	52%	67.3–78.8%	6–10% rearing male layers 23–29% DP chicken 51–57% in-ovo sex determination
Reithmayer et al. (39)	Dec-March 2019 Germany	26 min Online survey 26 questions	482, given a small financial incentive	N.S.	89%	N.D. rearing male layers N.D. DP chicken Different in-ovo sex determination techniques: 48%
Reithmayer and MuBhoff (38)	2018 Germany	Online survey, 4 parts, DCE^d^, information part, questions	400, given a financial incentive	N.D.	N.D.	27% DP chicken 0% rearing male layers 75% in-ovo sex determination
Busse et al. (10)	2016 Germany	20-min telephone interviews, 43 questions	1,000 consumers of an organic farming initiative	70%	67%	N.D. Rearing male layers 50% DP chicken N.D. in-ovo sex determination
Gremmen et al. (36)	Oct-Dec 2015 The Netherlands	Online survey^a^, 10 questions, 2 blocks of informative text,	1,022^b^	55%	47%	41.3% DP chicken 41.3% rearing layer males 37.5–43.2%^c^ pref. in-ovo sex
Gangnat et al. (40)	Jan-Feb 2016 Switzerland	10-min survey on DP, at 8 supermarkets, 18 questions, text, photos, ruler for WTP	402^e^, small gift	25%	N.D. % but preference for in-ovo sex determination over CMC	N.D. % alternatives N.D. Rearing male layers No preference for DP over CMC Preference for in-ovo sex determination over CMC
Leenstra et al. (9)	N.S. The Netherlands	Online survey^f^, film, 10 alternatives	1,199	42%	58%	24% DP chicken N.D. rearing male layers 25% in-ovo sex determination

* *Dataset divided in Highly Educated Women (n = 143) and REST (n = 85). N.S., Not specified; N.D., not determined;*

a *Survey made with use of a valorization panel: stakeholders (farmer representatives), consumers; retail and animal protection organizations, n = 10;*

b *overrepresentation of highly educated people and 50–69 age group;*

c *different types of in ovo sex determination were examined (genetic modification, invasive and non-invasive methods);*

d *DCE: discrete choice experiment; choose between 2 or more alternatives*,

e *Respondents were either given questions focussed on eggs or on chicken;*

f *Survey made with use of 6 focus groups with 6–7 people, tested on 44 students*.

## Discussion

In this study, we aimed to assess the awareness of the Dutch public at this point in time on the practice of as a common practice in the egg-producing industry. We also aimed to determine which alternative to CMC was most preferable and assess WTP for eggs and cockerel burgers without CMC. However, probably due to the sampling technique (distribution *via* social media channels of the authors), our sample distribution was biased toward a select subset of Dutch society.

### Data

The data cannot be seen as representing the Dutch population. Due to this limitation, we have been careful not to overinterpret the sociodemographic aspects of our results. For both subsets, we had an underrepresentation of people with a low income and an overrepresentation of participants from the provinces of Utrecht and Gelderland, which was likely to be because we used personal connections of the authors for our survey. For the future, distribution *via* other sources is recommended [i.e., *via* egg producers (10), survey companies (9, 36), at supermarkets (40)] to obtain a more balanced view of the Dutch population and their buying habits. However, even though respondents do not represent the Dutch population, and subset REST was more diverse than subset HEW, and the result of both subsets were overall very similar. It appears that our respondents fit the targeted consumer class of “Price-insensitive In-Ovo supporters” (10, 38) as cost (WTP) was the least important factor for choice of alternatives to CMC. Our results should be a trigger to do further research on this topic in a broader range of the Dutch society to include other classes of consumers (10, 38). The setup of our study could be used in future surveys on CMC by giving insight on which factors to include, i.e., provide information on the poultry industry and including more elaborate information on the alternatives with regard to sustainability and economics.

A further explanation for the skewed respondents' background might be due to the topic of the questionnaire and the ethical issues it raises. People may feel uneasy knowing that the CMC takes place for the production of eggs and prefer to remain blind to the subject [i.e., a moral lock-in; (31)]. This might explain the high number of respondents initially starting the questionnaire but not finishing it (259 out of 372).

### Awareness

Our study showed comparable awareness levels (52%) as in other studies in the Netherlands in 2018 [55%; (36)] and 2011 [55%; (9)] but higher than in Switzerland [25%; (40)], see Table 6. Awareness and disapproval of CMC show an increase in several EU countries (36). Highest awareness levels were found in the provinces where most chicken farms are present in the Netherlands^1^. The rise in awareness from 2011 to 2018 in the Netherlands was accredited to media attention in 2013 caused by a study commissioned by The Dutch State Secretary of Agriculture on public views on alternatives for CMC (36). In France and Germany, a rise in public awareness on CMC likely caused pressure to ban CMC and seek viable alternatives.

### Alternatives to Culling Male Layer Chicks

A very high percentage of our respondents agreed that an alternative to CMC is needed. Keeping the current situation was also less preferred as opposed to a study in 2018, where 28% chose keeping CMC (36). The first choice for an alternative for CMC was *in-ovo* sex determination, by 57% of the respondents, which is higher than that in other studies (9, 36). In our study, we only looked at alternatives that are available on the Dutch market and did not distinguish between types of *in-ovo* sexing techniques and at which embryonic stage the *in-ovo* technique is applied (3) as Leenstra et al. (9) did. They showed that *in-ovo* sex determination was most preferable, but only on non-incubated eggs. With this method, the sex of the embryo is determined in the fresh egg, and male eggs are excluded from incubation as opposed to determining the sex of the embryo in incubated eggs during early or late development (9). An aspect to consider in *in-ovo* sex determination that people may choose or not choose this alternative to CMC is at what age it takes place (39, 42, 43). Studies on neural development of chick embryos (44, 45) indicate that afferent nerves develop around day 4 of incubation and that the synaptic connection *via* the spinal cord is not present before day 7 of incubation, which make nociception impossible prior to this stage. At what age *in-ovo* sex determination is performed should be included (and explained in the survey), as it does influence respondents' attitude to *in-ovo* sexing (38). In another study in Dutch citizens, *in-ovo* sex determination was only accepted by 11% of respondents (36). In Switzerland, no difference was found between *in-ovo* sexing and keeping dual-purpose chicken as an alternative to CMC (40). An informed decision for an alternative requires knowledge of the pros and cons of the alternative and the current practice (9). Underlying social norms and values may overrule the initial choice (10) for an alternative. At the same time, when more information is provided about the alternatives, the decision for one or the other may not be so easy (9). Zoll et al. (11) developed a decision support tool for the case of a dual-purpose alternative to CMC in Germany, which could be used when targeting the Dutch society. Based on our outcome and the results of surveys in the past in the Netherlands and other EU countries, it appears that the majority of participants in these studies respond to the knowledge on CMC by disagreement and are strongly in favor of an alternative to CMC.

### Welfare Cockerels

Based on our results, the respondents indicated that at this point in time, *in ovo* sex determination is more likely to be chosen as an alternative to CMC over rearing male layers and keeping dual-purpose chickens. It should be noted that aspects of sustainability and economics were absent specifically from our study, and that the choice of alternative should be under the highest level of welfare. Studies on keeping layer males (15) or dual-purpose chicken (13, 46, 47) are relatively scarce and require more investigation as to whether and how these cockerels can be kept under high as there might be issues with aggression (48).

Economic incentives are needed to pursue this avenue also with respect to sustainability issues since it takes longer to raise cockerels, which require more food and other costs, as opposed to broiler chicken. In Thailand, layer males are kept for 60 days to achieve a body weight of 0.8–1.2 kg (49). In a growing world population where broiler chicken meat production is more sustainable than other sources of animal protein, it makes the choice for other—less profitable (50, 51)—chicken meat difficult. Additionally, the meat of layer cockerels has a different texture and taste from those of broiler meat (52). In Asia and Africa, however, meat of cockerels of layers and native chicken is very popular (53, 54).

### Willingness to Pay

Respondents were willing to pay [willingness to pay (WTP)] more for eggs without CMC involved even above the current price for existing CMC-free eggs [Kipster® (www.kipster.nl: keeping male layers for meat products); Respeggt (www.respeggt.com: *in-ovo* sex determination and exclusion of male embryos for further development)]. Price per egg on the Dutch market varies by type of farming system the hens are kept as well as the type of hen (i.e., brown or white). The price of eggs fluctuates due to seasonal changes, demand and supply, and legal restrictions (i.e., keeping hens indoors during bird flu). At present, the price per egg (approximation over different Dutch supermarkets; 2021) from white barn layers is €0.16, from brown barn layers is €0.20, free-range white or brown layers is €0.29 and for organic layers is €0.45. Currently, Dutch Kipster eggs are sold (at a specific supermarket) for €0.25 per egg and German Respeggt eggs are being sold for €0.39 per egg. Most of the respondents who consumed eggs were WTP at least or more than €0.35 per egg without CMC. This is consistent with earlier studies, showing that 50–60% of respondents were WTP 5–10 cents more per egg (9). Although our respondents were less inclined to buy processed poultry meat products, they are WTP at least €3.50 for two processed cockerel burgers, 1€ more than the current price of cockerel burgers in the Netherlands. However, it should be mentioned that a large percentage of our respondents (HEW: 28.7%; REST: 36.0%) had a relatively high income (>€50.000, income levels were similar to those by Dutch statistical database; statline.nl), which may have skewed this result. Furthermore, no relationship was found between the importance of price and income level. This also supports the aforementioned idea that our respondents who buy meat and eggs fit within the “Price-insensitive In Ovo supporters”—a specific class of consumers, and the results should be viewed as biased toward this type of consumers (38). We did not ask how often meat or eggs are bought as done by Gangnat et al. (40) and Busse et al. (10), and thus, we cannot fully assess the motives of our respondents, and further research is needed to understand which factors influence choice for specific poultry products. One issue in surveys is whether the WTP given is the real WTP of consumers (55), i.e., the price people indicate to pay and the real price they are paying. Therefore, future research on this topic should include indirect measures of WTP and estimate a hypothetical bias that is higher for niche products, such as eggs without CMC and cockerel meat.

### Factors for Choosing

The most important factor when buying poultry meat or eggs was food safety, similar to a study of the Dutch public in 2018 (36). Interestingly, this was not the first factor in earlier studies on the alternative for CMC under the Dutch public (9). All three studies [this study, (36), and (9)] have animal friendliness as an important factor for choice of alternative for CMC. In the study by Leenstra et al. (9), animal friendliness was the most important factor. Likely due to the provision of more in-depth understanding of the topic (i.e., by a film and focus group), respondents could make a better assessment on the factors of importance for this type of animal-based product. The image of culling day-old chicks may have also further shocked participants in that study, as it did regarding information on CMC in the survey in Switzerland (40). Naturalness was an important factor in alternatives to CMC (9) and when accepting specific foods and certain technologies involving food production (56). This aspect is consistent across countries and years, indicating that it might be one of the most important factors to include when promoting CMC-free food products. We did not explain or define naturalness and whether this factor is excluding or including aspects such as animal welfare and the environment. In future surveys, the definition of naturalness should be provided either by the respondents (i.e., in a preliminary questionnaire) or given by the survey makers.

### Informed Choice and Labels

Our results showed that providing respondents with information on the poultry industry decreased the level of accepting CMC, i.e., more disagreement after the questionnaire than before. This was comparable to Swiss consumers, who were more WTP when they knew more about the poultry industry (40), which is stipulated as important to make an informed choice in this matter (9). In our study, labels were preferred so as to make a choice for CMC-free products easier. These results were also found in other studies where labels were recommended (40). Pictures may provide shocking images but could also be helpful in communicating the essential ethical elements of CMC (57) and the process of *in-ovo* sex determination techniques in detail to establish a higher WTP (58).

## Conclusion

This study aimed to assess awareness and acceptability of the practice of CMC of consumers in the Netherlands. Our results should be viewed as a pilot where we examined the opinion of a selective population of respondents toward CMC and its alternatives. Interestingly, providing information on the poultry industry during the course of the survey influenced acceptability (i.e. not accepting) of CMC. More detailed information on the alternatives with regard to economic, sustainability, and ethical factors is needed for respondents to assess their preference for alternatives and poultry products with or without CMC. A follow-up study on this topic with data from a more diverse population in the Dutch industry could be further improved/targeted using our results to help assess this complex issue within the egg industry.

## Data Availability Statement

The raw data supporting the conclusions of this article will be made available by the authors, without undue reservation.

## Author Contributions

EdH idea and writing manuscript. EO conducted the research. MvG design survey. All authors contributed to the article and approved the submitted version.

## Conflict of Interest

The authors declare that the research was conducted in the absence of any commercial or financial relationships that could be construed as a potential conflict of interest.

## Footnotes

^1^https://www.cbs.nl/
