# Supplementary material for: The Need for an Alternative to Culling Day-Old Male Layer Chicks: A Survey on Awareness, Alternatives, and the Willingness to Pay for Alternatives in a Selected Population of Dutch Citizens
Source: Front Vet Sci. 2021 Jun 17;8:662197. doi: 10.3389/fvets.2021.662197 (PMC8248538; doi:10.3389/fvets.2021.662197)
Supplement: Supplementary file 1 [file Table_1.doc]

**SUPPLEMENTARY INFORMATION**

**Questionnaire of survey on culling day old male layer chicks (CMC)**

**Paragraph 1.** **Knowledge on the poultry industry** *with correct answer between ()*

**1. How many chickens are kept in The Netherlands yearly?**

A. less than 10.000.000; B. Between 20.000.000 and 40.000.000; C. Between 40.000.000 and 80.000.000; D. More than 80.000.000 (**D**)

**2. At which age are chickens kept for meat being slaughtered?**

A: Between 5 and 7 weeks of age; B. Between 16 and 17 weeks of age; C: between 38 and 40 weeks of age; D. Between 80 and 100 weeks of age (**A**)

**3. Of all fertilized eggs the sex is being determined of the embryo.**

A: True; B: False (**B**)

**4. After hatching, the sex of the chick is being determined manually by a specialist.**

A: True; B: False (**A**)

**5. All cockerels, from meat and layer chickens, are culling as day old chicks in the hatchery.**

A: True; B: False (**B**)*

**6. Only cockerels of layer chickens are culled as day old chicks in the hatchery.**

A: True; B: False (**A**)

**7. Culling of day old chicks is conducted via CO_2_ gassing.**

A: True; B: False (**A**)

**8. Culled day old chicks are used as feed for animals in zoos, reptiles, birds of prey and other animals.** A: True; B: False (**A**)

**Paragraph 2.** **Acceptance of culling day old male layer chicks**

Agreement questions levels of agreement:

A: Totally disagree, B: Disagree, C: neutral, D: Agree, D: Totally agree

**9. Culling day old male layer chicks is a good solution.**

**10. Culling day old male layer chicks is not a problem.**

**11. Culling day old male chicks is unavoidable.**

**12. There is a need for an alternative to culling day old male layer chicks.**

**Paragraph 3. Acceptance of alternatives to culling day old male layer chicks**

Agreement questions levels of agreement:

A: Totally disagree, B: Disagree, C: neutral, D: Agree, D: Totally agree

**13. Chickens can be kept for the production of human food.**

**14. Day old male layer chicks can be culled.**

**15. Because culled day old male layer chicks have another purpose after death, this makes the need for an alternative to culling day old male layer chicks unnecessary.**

**16. The use of a double purpose chicken is a good alternative to culling day old male layer chicks.**

**17. Keeping layer cockerels for their meat is a good alternative to culling day old male layer chicks.**

**18. Sex determination, and excluding male embryos, is a good alternative to culling day old male layer chicks.**

**Paragraph 4. Preference of alternatives to culling day old male layer chicks**

Preference levels:

1: 1^st^ preference, 2: 2^nd^ preference, 3: 3^rd^ preference, 4: 4^th^ preference, 5: 5^th^ preference

**19. Assign preference levels for**

**__ Accepting the culling day old male layer chicks;**

**__ Keeping the layer cockerels for a longer period of time and slaughtering them for their meat;**

**__ Using a double purpose chicken, instead of using broiler chicken for meat, and layer chicken for eggs)**

**__ Determination of sex in the egg and excluding male embryos**

**Paragraph 5. Factors of importance affecting choice when buying chicken products**

When choosing chicken meat which factors do you find important?

Importance levels:

A: Very important, B: Important, C: neutral, D: Important, D: Very important, E: Not applicable

**20. Price**

**21. Environment**

**22. Availability of the product**

**23. Food safety**

**24. Naturalness**

**25. Animal friendliness**

**26. Taste**

**27. Feasibility of the alternative**

**Paragraph 6. Willingness to pay for chicken products**

**28. Which type of eggs do you buy?**

A: Organic, B: Free range, C: Barn, D: Kipster*, D: Other (not Kipster or Organic) 3-star animal welfare labelled, E: Not applicable

* Kipster is explained, Kipster is a chicken farm where layer cockerels are kept in addition to keeping the layer chickens of a commercial Dekalb White layer breed

**29. How much are you willing to pay for 10 eggs?**

A: 2,00€, B: 2,50€, C:3,00€, D: 3,50€, D: 4,00€, E: 4,50€, F: 5,00€, G: Not applicable

**30. How much are you willing to pay for 10 eggs where no culling of male layer chicks have taken place?**

A: 2,00€, B: 2,50€, C:3,00€, D: 3,50€, D: 4,00€, E: 4,50€, F: 5,00€, G: Not applicable

**31. Which type of chicken meat do you buy?**

A: Chicken breast, B: Chicken legs excluding the thighs, C: Chicken thigh, D: processed chicken meat such as burgers or nuggets, E: Not applicable

**32. How much are you willing to pay for two cockerel burgers?**

A: 1,50€, B: 2,50€, C:3,50€, D: 4,50€, D: 5,00€, E: Not applicable

**33. Do you use a welfare label when buying chicken products?**

A: yes, B: no, C: not applicable

**Paragraph 7. Acceptability of CMC**

**34. During the course of this survey we have provided you with information on the culling of day old male layer chicks. To which extent are you agreeing with this practice?**

A: Totally disagree, B: Disagree, C: neutral, D: Agree, D: Totally agree
